# Wild-type menin is rapidly degraded via the ubiquitin-proteasome pathway in a rat insulinoma cell line

**DOI:** 10.1042/BSR20190471

**Published:** 2019-10-18

**Authors:** Zongzhe Jiang, Shengrong Wan, Bowen Xing

**Affiliations:** 1 Experimental Medicine Center, The Affiliated Hospital of Southwest Medical University, Luzhou 646000, Sichuan, China; 2 Endocrinology Department, The Affiliated Hospital of Southwest Medical University, Luzhou 646000, Sichuan, China; 3 Diabetes Research Center, School of Medicine, Shenzhen University, Shenzhen 518060, Guangdong, China

**Keywords:** INS-1 cell, Insulinoma, Menin, Ubiquitin-proteasome

## Abstract

Menin is encoded by multiple endocrine neoplasia type 1 (*MEN1*) gene, the germ line mutations of which are the main cause of pancreatic neuroendocrine tumors (PNETs). To date, a large number of frameshift, nonsense and missense mutations of *MEN1* have been identified to be responsible for part of *MEN1*-defficient PNETs patients due to truncation or rapid degradation of menin protein. However, the stability of the wild-type (WT) menin in PNETs is totally unknown. In the present study, we observed ubiquitination of WT menin in 293T cells by transfection of ectopic WT menin and HA-ubiquitin. As expected, either endogenous or ectopic WT menin is stable in 293T cells, whereas in INS-1 cells, a rat insulinoma cell line derived from PNETs, either endogenous or ectopic WT menin is rapidly degraded through ubiquitin-proteasome pathway. Furthermore, the degradation of WT menin is more rapid in the presence of serum. Our findings suggest that in part of PNETs patients with WT *MEN1*, a ubiquitin-proteasome system targeting menin is untimely activated.

## Introduction

Pancreatic neuroendocrine tumors (PNETs) arise from the endocrine cells of the pancreas which are also known as the islets of Langerhans [1]. Some PNETs occur in patients with multiple endocrine neoplasia type 1 (MEN1) [2], identified by the presence of neoplasms in two or more different endocrine tissues such as parathyroid glands, endocrine pancreas and anterior pituitary gland [3]. The *MEN1* gene encodes for the protein menin and individuals with MEN1 mutations will develop MEN1 [1]. As reported by Papadopoulos et al. in 2011, 44% of the PNETs had somatic inactivating mutations in *MEN1* [4], indicating a strong correlation between menin-deficiency and the development/progression/initiation of PNETs.

Menin, an evolutionarily conserved and ubiquitously distributed scaffold protein, has functions in multiple physiological processes. Menin has been shown to function in DNA repair, cell cycle and cell structure through interacting with FANCD2 [5], the 32-kDa subunit (RPA2) of replication protein A [6] and GFAP and vemintin [7]. In addition, Menin can interact with a number of transcriptional activators including protein-energy malnutrition (Pem) [8], Hlxb9 [9] and mixed lineage leukemia proteins (MLL) [10] to activate the expression of tissue-specific target genes and recruit histone modifiers including histone deacetylase (HDAC)1/2 [11], Sirt1 [12], the histone H3 lysine 27 methyltransferase EZH2 [13], and protein arginine methyltransferase (PRMT) 5 [14] to repress the transcription of target genes. As a tumor suppressor, the deletion of *MEN1* in mouse pancreatic β cells results in the formation of functional PNETs (insulinomas) after 6 months of age [15], suggesting that menin is essential for the control of β-cells proliferation.

Although majority of the deficiency of encoded protein menin in PNETs is caused by abnormal transcripts from inactivating mutations of *MEN1* [16] or the rapid degradation of missense mutants via the ubiquitin-proteasome pathway [17,18], some PNETs with wild-type (WT) sequence of *MEN1* also showed a lower level of menin expression in menin immunohistochemical staining [19]. Furthermore, it is unknown that whether the menin in PNETs with WT *MEN1* is also unstable. In the present study, we observed ubiquitination of WT menin *in vivo* and first reported the rapid degradation of endogenous or ectopic WT menin in one of PNET-derived cell line.

## Materials and methods

### Cell lines and cell culture

Stable Flag-Menin-expressing INS-1 cells were established by transduction with pMX-puro-Menin and RetroQ-puro-Shmen1-derived retroviruses, as previously reported by Feng et al. [20]. 293T cells were cultured in Dulbecco’s Modified Eagle’s Medium (HyClone) supplemented with 10% FBS and 1% Pen/Strep. INS-1 cells were cultured in RPMI 1640 (Gibco) supplemented with 10% FBS, 1 mol/l Hepes, 0.2 mol/l l-glutamine, 0.1 mol/l Sodium pyruvate, 55 mmol/l β-Mercaptoethanol.

### Plasmids

WT full-length menin was amplified by PCR and cloned into the BamHI/NotI site of pCDNA3.1. Retroviral plasmid pMX-puro-menin was constructed by inserting PCR-amplified Menin cDNA into the BamHI/NotI site of the retroviral vector pMX-puro.

### Plasmids transfection

Transfection of Flag-Menin and/or HA-Ubiquitin was performed according to the typical Lipofectamine 2000 transfection procedure. Briefly, diluted 10 μg DNA with 1 ml Opti-MEM Medium and mixed with 1 ml diluted Lipofectamine 2000 Reagent. The mixture was incubated for 5 min at room temperature and DNA–lipid complex was added into 293T cells afterward.

### Immunoprecipitation

For immunoprecipitation, cells were suspended in lysis buffer (50 mmol/l Tris/Cl, pH 7.4, 150 mmol/l NaCl, 5% glycerol, 1% NP-40, 1 mmol/l EDTA), supplemented with 1 mM PMSF, 4 μg/ml protease inhibitor cocktail (Sigma). Lysates were centrifuged at 13000×***g*** for 10 min, and the supernatant was added to 2 μl indicated antibodies and 100 μl Protein A agarose (Invitrogen) to incubate for 4 h at 4°C. Afterward, Protein A agarose was washed by 250 mmol NaCl for four-times.

### Western blotting

For Western blotting, cells were collected at indicated time points and then were lysed by RIPA lysis buffer (Beyotime, Nantong, China). Cell lysates (90 μl) were mixed with 30 μl SDS loading buffer and boiled for 5 min at 100°C for SDS/PAGE. Primary antibodies were diluted according to instructions. HRP-labeled secondary antibody was used at a dilution of 1:3000. Immuno-reactive bands were revealed by enhanced chemiluminescence (Clarity™ Western ECL Substrate, Bio-Rad) and visualized by the Image Quant LAS 4000 mini (GE). Band intensities were quantified and analyzed with ImageJ and normalized against the level of β-actin.

### Chemicals and inhibitors

MG132 was purchased from Sigma–Aldrich and dissolved in DMSO. Cycloheximide (CHX) was purchased from AMRESCO and dissolved in DMSO.

### Antibodies

Antibodies used were Rabbit polyclonal anti-menin (Bethyl, A300-105A), mouse monoclonal anti-HA (CWbiotech, CW0092A) and mouse monoclonal anti-β-actin (Santa Cruz, SC47778).

### Statistical analysis

Data are presented as mean ± S.E.M. for the indicated number of experiments (*n*). Statistical significance was evaluated using the Student’s *t* test. Data were considered significant when *P*<0.05.

## Results

### WT menin can be ubiquitinated *in vivo*

**Figure 1:**
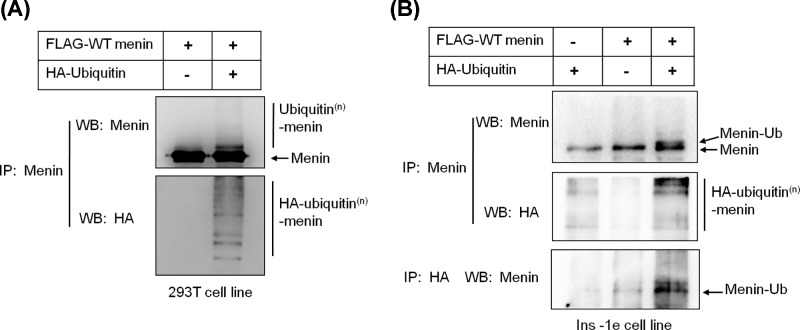
WT Menin can be modified with ubiquitin in 293T cells (**A**) Equal amounts of Menin and/or HA-ubquitin constructs were transfected into 293T cells, followed by MG132 (20 μmol) treatment for 4 h and lysed for IP and Western blot with the indicated antibodies. (**B**) Equal amounts of Menin and/or HA-ubquitin constructs were transfected into INS-1 cells, followed by MG132 (20 μmol) treatment for 4 h and lysed for IP and Western blot with the indicated antibodies.

To determine whether WT menin could be ubiquitinated, we performed an *in vivo* ubiquitination assay. 293T cells were co-transfected with constructs expressing HA-ubiquitin and FLAG-tagged WT menin or WT menin only, and lysates were immunoprecipitated with an anti-menin antibody. Western blotting with anti-menin and anti-HA antibodies respectively detected menin-ubiquitin bands (Figure 1A), indicating WT menin is ubiquitinated *in vivo*. Given that the mutant menin could be degraded in MEN1, we also performed an *in vivo* ubiquitination assay of WT menin in an insulinoma cell line, INS-1. The result showed that WT menin was also ubiquitinated in the INS-1 cells (Figure 1B).

### WT menin is stable in 293T cells

**Figure 2:**
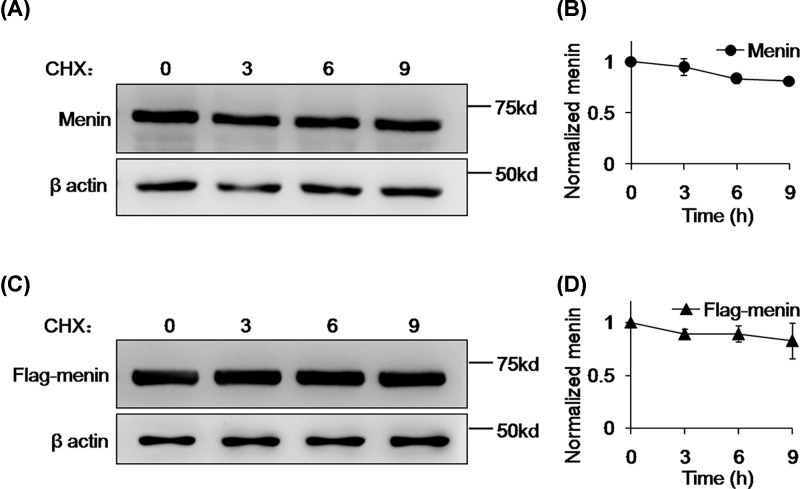
Menin is stable in 293T cells (**A**) 293T cells were treated with CHX (20 μg/ml) for the indicated times and lysed for Western blot. (**B**) Quantitation of menin protein level from (A). Gray analysis was executed with ImageJ and menin protein levels were quantified and normalized to β-actin. (**C**) 293T cells transfected with Falg-menin were treated with CHX (20 μg/ml) for the indicated times and lysed for Western blot. (**D**) Quantitation of the Flag-menin protein level from (C). Gray analysis was executed with ImageJ and Flag-menin protein levels were quantified and normalized to β-actin.

As the WT menin could be modified with ubiquitin *in vivo*, we next estimated the stability of WT menin in 293T cells with CHX treatment. Whole-cell extracts from 293T cells treated with CHX for indicated times were subjected to Western blotting and the clearance rate of WT menin was monitored by Western blot analysis. The protein level of WT menin from indicated CHX treatment exhibited no obvious difference in Western blot bands (Figure 2A) and the clearance rate was no more than 20% after 9-h CHX treatment (Figure 2B). This result was confirmed by CHX chase analysis of the ectopic FLAG-tagged WT menin. 293T cells transfected with FLAG-tagged WT menin were treated with CHX for indicated times and subjected to Western blotting. As shown in Figure 2C,D, FLAG-tagged WT menin was also stable at protein level and clearance rate in 293T cells.

### WT menin is degraded rapidly in INS-1 cells

**Figure 3:**
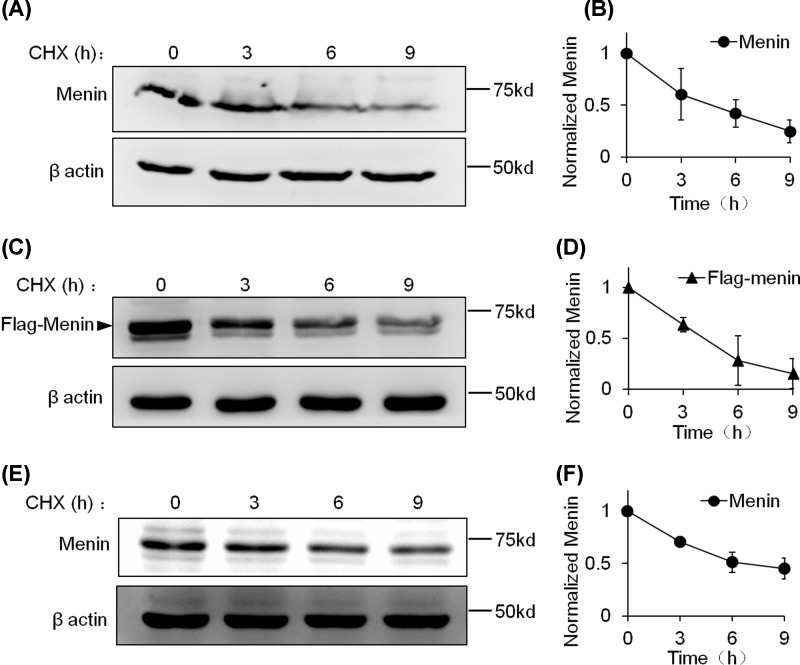
WT menin degrades rapidly in the presence of CHX in INS-1 cells (**A**) INS-1 cells were treated with CHX (20 μg/ml) for the indicated times and lysed for Western blot. (**B**) Quantitation of menin protein level from (A). Gray analysis was executed with ImageJ and menin protein levels were quantified and normalized to β-actin. (**C**) INS-1 cells expressing ectopic Falg-menin were treated with CHX (20 μg/ml) for the indicated times and lysed for Western blot. (**D**) Quantitation of the Flag-menin protein level from (C). Gray analysis was executed with ImageJ and Flag-menin protein levels were quantified and normalized to β-actin. (**E**) TGP-61 cells were treated with CHX (20 μg/ml) for the indicated times and lysed for Western blot. (**F**) Quantitation of the menin protein level from (E). Gray analysis was executed with ImageJ and menin protein levels were quantified and normalized to β-actin.

Given that the WT menin is stable in 293T cells, we turned to investigating the stability of WT menin in pathological cells, such as PNETs. To this end, we employed INS-1 cell, a rat insulinoma cell line, to estimate the degradation rate of WT menin. Whole-cell extracts from INS-1 cells treated with CHX for indicated times were subjected to Western blotting and the clearance rate of WT menin was monitored by Western blot analysis. In contrast with the stability in 293T cells, we observed rapid degradation of WT menin in INS-1 cells (Figure 3A). After CHX treatment for 9 h, nearly 80 percent of WT menin was degraded and the half-life of WT menin was estimated to be approximately 4 h in INS-1 cells (Figure 3B). To confirm the rapid degradation of WT menin in INS-1 cells, we constructed INS-1 cell line stably expressing ectopic FLAG-tagged WT menin which was the same as the one we used in 293T cells. Similarly, we observed significant degradation of FLAG-tagged WT menin in ectopic menin-expressing INS-1 cells (Figure 3C,D). To confirm that the WT menin can be degraded in insulinoma cells, we employed another insulinoma cell line, TGP-61, to detect the stability of WT menin. The result showed that WT menin was also significantly degraded in TGP-61 cell line (Figure 3E,F). These findings suggest that WT menin reduction resulting from rapid degradation is the possible cause of PNETs in part of patients who have normal *MEN1* gene.

### WT menin is targeted for degradation via the ubiquitin-proteasome pathway in INS-1 cells

**Figure 4:**
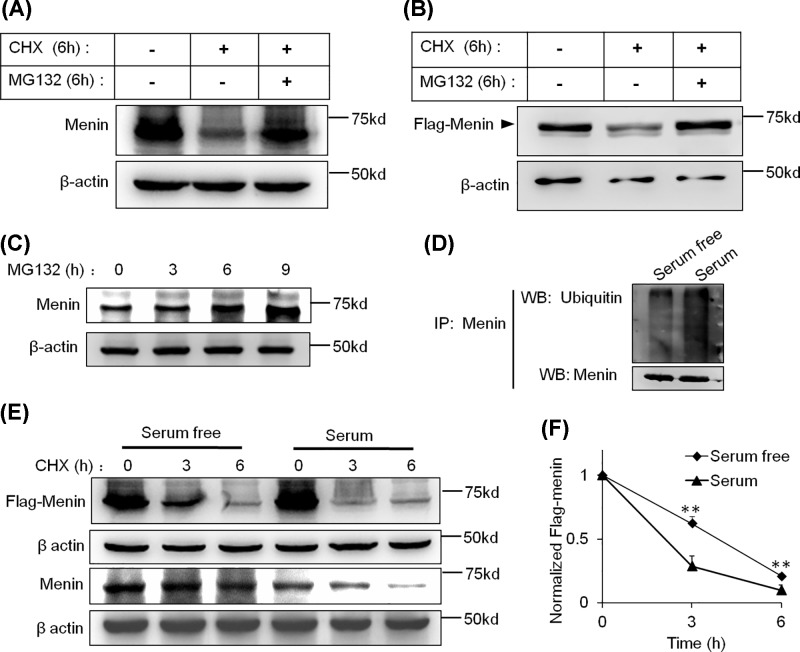
Degradation of WT menin could be blocked by MG132 in INS-1 cells (**A**) INS-1 cells were treated with CHX (20 μg/ml) and MG132 (20 μmol) or CHX (20 μg/ml) only for 6 h and lysed for Western blot. (**B**) INS-1 cells expressing ectopic Flag-menin were treated with CHX (20 μg/ml) and MG132 (20 μmol) or CHX (20 μg/ml) only for 6 h and lysed for Western blot. (**C**) INS-1 cells were treated with MG132 (20 μmol) for the indicated times and lysed for Western blot. (**D**) INS-1 cells were treated with MG132 (20 μmol) for 4 h and lysed for IP and Western blot with the indicated antibodies. (**E**) INS-1 cells or INS-1 cells expressing ectopic Flag-menin were treated with CHX (20 μg/ml) for the indicated times in the presence or absence of serum and lysed for Western blot. (**F**) Quantitation of Flag-menin protein level from (E). Gray analysis was executed with ImageJ and Flag-menin protein levels were quantified and normalized to β-actin. **, *P*<0.01.

In eukaryotic cells, the ubiquitin-proteasome pathway is one of the major mechanisms for the targeted degradation of unstable proteins. Therefore, we investigated the effects of the proteasome inhibitor MG132 on the stability of WT menin during CHX addition to confirm whether WT menin is targeted for degradation via the ubiquitin-proteasome pathway in INS-1 cells. As shown in Figure 4, whether the amount of WT menin (Figure 4A) or FLAG-tagged WT menin (Figure 4B) could be restored by MG132 treatment in the presence of CHX in INS-1 cells. Especially, MG132 treatment alone significantly accumulated the amount of WT menin (Figure 4C). These data suggest that the ubiquitin-proteasome pathway was implicated in the rapid degradation of WT menin.

### WT menin is degraded slowly in serum-free medium

To further investigate the regulation of WT menin degradation in INS-1 cells, we performed CHX chase analysis with removal of the serum from medium. INS-1 cells expressing ectopic WT menin were serum-starved overnight and then incubated with or without 10% FBS and CHX for indicated times. Whole-cell extracts were analyzed by IP with menin antibody and Western blotting with menin and ubiquitin antibodies. The result showed that there was more ubiquitination of menin in the presence of serum than that in the absence of serum (Figure 4D). For further confirmation, the clearance rate of WT menin was monitored by Western blot analysis. As shown in Figure 4E, the protein level of WT menin (Flag-menin or endogenous menin) at 3-h CHX treatment in serum-free medium was obviously higher than that in serum medium, which was also confirmed in clearance rate (62 vs 29%; Figure 4F). These results were further confirmed by the fact that serum-free culture led to a little reduction in menin mRNA, but with no significance (Supplementary Figure S1A), indicating that some factors in serum might contribute to the ubiquitin-proteasome pathway mediated degradation of WT menin.

## Discussion

To date, more than 1500 different somatic and germline *MEN1* gene mutations have been identified [21,22]. Most of the studies focused on the truncated forms of nonsense mutations and the rapid degradation of missense mutations of MEN1 [17,18], whereas few researchers investigate the stability of WT menin even though some PNETs with WT MEN1 gene show lower protein level of menin. Here, we reported that the WT menin can be ubiquitinated but is very stable in 293T cells (Figures 1 and 2) and for the first time, we reported the rapid degradation of WT menin mediated by ubiquitin-proteasome pathway in a PNET-derived cell line, INS-1 (Figures 3 and 4).

**Figure 5:**
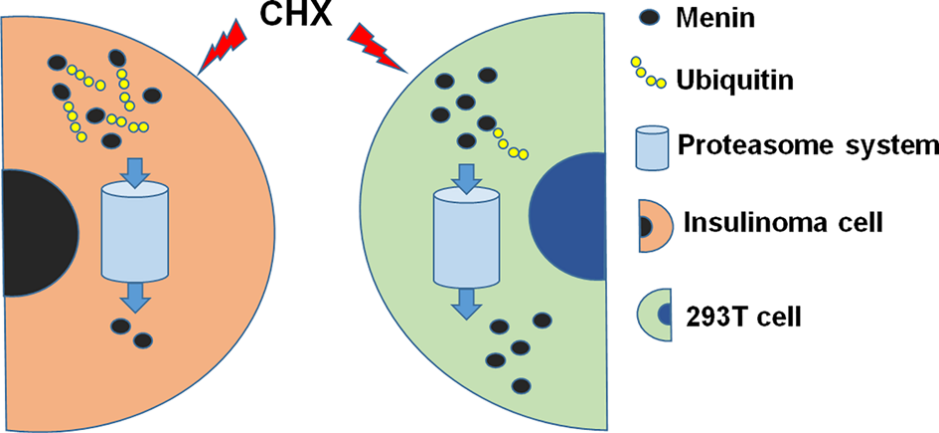
Schematic diagram demonstrates a new regulatory pathway of WT menin in insulinoma cells .

The finding that WT menin is very stable in 293T cells is also consistent with the results reported by Tsukada et al. [17]. In their studies, whether transiently expressed FLAG-tagged WT menin or endogenous menin is stable in 293T cells [17]. In some PNETs with WT *MEN1*, the protein level of menin in nucleus was reduced and the cytoplasmic staining was also absent, which excluded the possibility that menin was transported from nucleus to cytoplasm [19]. This finding indicates a potential transcriptional repression or protein degradation system targeting WT menin in PNETs with WT *MEN1* gene. As a rat insulinoma cell line, INS-1 cell is quite an ideal cell model to study the stability of WT menin in PNETs. With this cell model, we observed obvious degradation of WT menin *in vivo*. Similar to the degradation of missense mutations reported previously [17,18], the degradation of WT menin is also mediated by ubiquitin-proteasome system. However, unlike the interaction of missense mutations with CHIP, one of the E3 ligases, to initiate the degradation, Co-IP assay showed no interaction of WT menin with CHIP (Supplementary Figure S1B) in the INS-1 cells, suggesting that the ubiquitin-proteasome mediated degradation of WT menin in INS-1 is dependent on another E3 ligase. This E3 ligase might mediated ubiquitin-proteasome system to degraded WT menin only in the insulinoma cells (for example, INS-1 or TGP-61), but not non-insulinoma cells (for example, 293T cells). In a word, our studies propose a new regulation pathway of WT menin in PNETs (Figure 5).

## Supplementary Material

Supplementary Figure S1

## Abbreviations

CHIP: carboxylterminus of the hsp70 interacting protein
CHX: cycloheximide
Co-IP: co-immunoprecipitation
EZH2: enhancer of zeste homolog 2
FANCD2: fanconi anemia group D2
GFAP: glial fibrillary acidic protein
HA: hemagglutinin
Hlxb9: H2.0-like homeobox b9
HRP: horseradish peroxidase
IP: immunoprecipitation
MEN1: multiple endocrine neoplasia type 1
Opti-MEM: optimal minimal essential medium
PNET: pancreatic neuroendocrine tumor
RIPA: radio immunoprecipitation assay
RPA2: replication protein A2
WT: wild-type
