# Supplementary material for: Wild-type menin is rapidly degraded via the ubiquitin-proteasome pathway in a rat insulinoma cell line
Source: Biosci Rep. 2019 Oct 18;39(10):BSR20190471. doi: 10.1042/BSR20190471 (PMC6822493; doi:10.1042/BSR20190471)
Supplement: Supplementary Figure S1 [file BSR-2019-0471_supp.pdf]

A

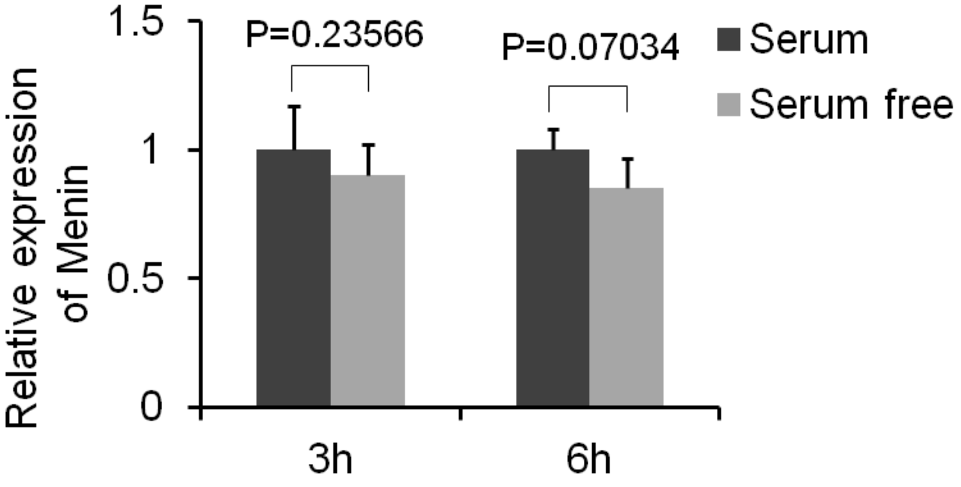

B

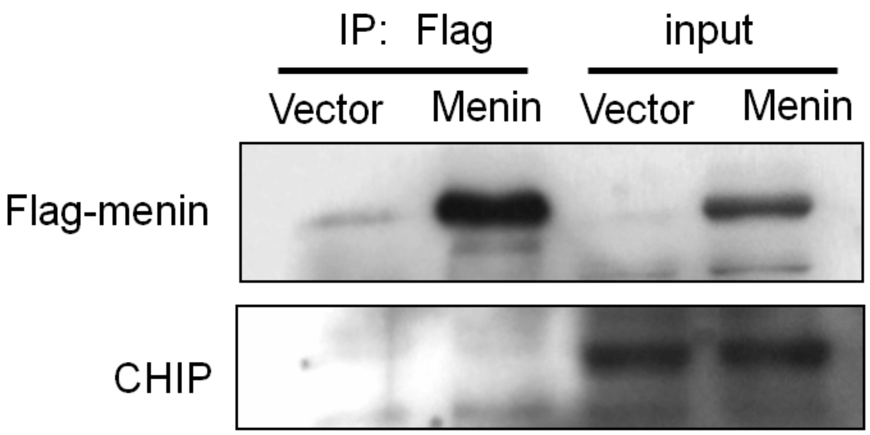

Supplemental Figure legend

Figure 1A. INS-1cells were treated with cycloheximide (20μg/ml) for indicated time and lysed for qRT-PCR to detect the mRNA level of *MEN1* gene.

Figure 1B. INS-1cells expressing ectopic Falg-menin were treated with MG132 (20 μmol) for 4 h and lysed for IP and Western blot with the indicated antibodies.
